# Fuzzy System to Assess Dangerous Driving: A Multidisciplinary Approach

**DOI:** 10.3390/s22103655

**Published:** 2022-05-11

**Authors:** Carlos Javier Ronquillo-Cana, Pablo Pancardo, Martha Silva, José Adán Hernández-Nolasco, Matias Garcia-Constantino

**Affiliations:** 1 Academic Division of Information Science and Technology, Juarez Autonomous University of Tabasco, Cunduacan 86690, Tabasco, Mexico; 191H18003@egresados.ujat.mx (C.J.R.-C.); martha.silva@ujat.mx (M.S.); adan.hernandez@ujat.mx (J.A.H.-N.); 2 School of Computing, Ulster University, Jordanstown BT37 0QB, UK; m.garcia-constantino@ulster.ac.uk

**Keywords:** AHP, dangerous driving, driver behavior, Dula dangerous driving index, fuzzy systems, intelligent transportation systems

## Abstract

Dangerous driving can cause accidents, injuries and loss of life. An efficient assessment helps to identify the absence or degree of dangerous driving to take the appropriate decisions while driving. Previous studies assess dangerous driving through two approaches: (i) using electronic devices or sensors that provide objective variables (acceleration, turns and speed), and (ii) analyzing responses to questionnaires from behavioral science that provide subjective variables (driving thoughts, opinions and perceptions from the driver). However, we believe that a holistic and more realistic assessment requires a combination of both types of variables. Therefore, we propose a three-phase fuzzy system with a multidisciplinary (computer science and behavioral sciences) approach that draws on the strengths of sensors embedded in smartphones and questionnaires to evaluate driver behavior and social desirability. Our proposal combines objective and subjective variables while mitigating the weaknesses of the disciplines used (sensor reading errors and lack of honesty from respondents, respectively). The methods used are of proven reliability in each discipline, and their outputs feed a combined fuzzy system used to handle the vagueness of the input variables, obtaining a personalized result for each driver. The results obtained using the proposed system in a real scenario were efficient at 84.21%, and were validated with mobility experts’ opinions. The presented fuzzy system can support intelligent transportation systems, driving safety, or personnel selection.

## 1. Introduction

Information systems based on fuzzy logic seek a classification result employing a rule-based inference engine. Such systems, known as fuzzy systems, aim to deal with the vagueness of human reasoning expressed linguistically by using formalisms. Linguistic expressions must be assigned a quantitative value that determines the group to which a variable belongs in the universe of discourse, so it is necessary to quantify the value of variables. This objectivity implies that electronic devices are often required for measurements so that values can be considered as objective [1].

However, there are many scenarios where users’ perceptions can be of great importance to feed the fuzzy system. For example, a diagnosis based on a doctor’s experience, determination of the quality of a product or service based on the opinions of customers, or people’s behavior assessment based on their self-evaluations. In such scenarios, the values of the variables can not be obtained using measurement equipment, thus it is necessary to use self-administered tools, such as questionnaires, for the participants to answer. Consequently, these are subjective values [2].

To our best knowledge, there is a lack of research in the literature about fuzzy system models that allow feeding a system combining objective and subjective values. We hypothesize that a fuzzy system with a multidisciplinary approach that combines objective and subjective variables can support solving complex social problems. Therefore, we propose a fuzzy system model where: (i) devices with sensors obtain data to feed the objective variables, and (ii) questionnaire responses that will provide values to the subjective variables of the system. We pre-processed the subjective data to quantify them as subjective variables and use them as input variables or in the inference rules.

Our fuzzy system model with multidisciplinary approach is composed of three-stages. The first stage consists of capturing objective data (numerical values) and subjective data (perceptions). The second stage is the processing of the objective and subjective data through two classification systems (OFS and SFS). Finally, the third stage consists of a fuzzy system where the classification results of objective and subjective data are combined, and a final output is obtained. The proposed model was applied to a case study on assessing dangerous driving resulting from negative cognitive/emotional, aggressive and risky driving.

This paper has two contributions. (1) A three-stage fuzzy system model with multidisciplinary approach that combines objective and subjective variables. The values of the objective variables are numerical and precise, while the subjective variables take values from human perceptions or opinions and can be ambiguous and imprecise. The reference model seeks to represent both the objective and subjective variables present in many classification scenarios. (2) A new way of assessing dangerous driving, which results from aggressive, risky driving behaviors and negative thoughts towards other drivers. The new assessment from implementing the reference model combines objective values obtained with electronic engineering sensors (acceleration, cornering, location and speed sensors) and subjective values (from a self-applied questionnaire on dangerous vehicle driving and another questionnaire on social desirability).

Questionnaires from behavioral science are useful to feed the fuzzy systems with user opinions and perceptions. Therefore, applying our model implies considering objective and subjective aspects to the evaluation of vehicular driving, making it more in line with and representative of the factors that may intervene during driving.

The remaining of the paper is organized as follows. Section 2 explains the background and related work. Section 3 provides the step-wise explanation of the proposed approach in detail. Section 4 and Section 5 present model implementation in the case study and report the obtained results, respectively. Section 6 discusses the experimental results. Finally, conclusions and final remarks are presented in Section 7.

## 2. Background and Related Works

### 2.1. Dangerous Driving Assessment

The dangerous driving of a vehicle can cause injuries to the driver, passengers and other people on the road, as well as economic, property and road infrastructure damages [3]. Given the above, some previous works in the literature have investigated how to determine the driver’s behavior during vehicle driving and how it contributes to decision making.

There are technological solutions that rely on in-vehicle sensor data to evaluate driving behavior. The CAN (Controller Area Network) bus is an example and has been used for safety and driver fingerprinting purposes [4,5]. The CAN bus is a communication system developed to exchange information between the electronic control units of an automobile. This system is factory installed in some vehicles and captures data from various types of sensors, though it has the disadvantage of being an expensive solution because it is contained in high-end vehicles. Some applications based on video camera data that employ artificial intelligence techniques are driver distraction [6] and driving style recognition based on vehicle trajectory [7] despite some dificulties of cameras usage such as ilumination changes, obstrusiveness and privacy issues.

Research works concerning the use of sensors in transportation systems are becoming more and more frequent. A great variety of experiments have analyzed vehicle movements during driving and aim to increase the safety and comfort of users [8]. Some authors have conducted experiments with a smartphone because it is an accessible, inexpensive tool with sensors (accelerometer, gyroscope, and GPS), which allows the analysis of vehicle movements [9,10,11]. Some of the variables that researchers have considered as input of artificial intelligence algorithms [12] are speed, braking, potholes, bumps, and environmental conditions [8,13,14].

Artificial intelligence techniques and methods employed to analyze vehicle movements obtained from sensors have proven to be efficient and effective. In the literature, some authors were able to classify driver behavior as dangerous, aggressive, risky (reckless), safe, unsafe, erratic and distracted, among others [15,16,17,18]. Evidence shows that the identification of driving styles for real scenarios is close to 80% [19]. Achieving higher efficiency levels is difficult due to erroneous reading caused by calibration failures [10,20].

In addition to the study of vehicle movements, the thoughts and actions of drivers can be analyzed to carry out a classification of driving behavior. Some of the tools used to gather subjective variables include questionnaires, interviews and self-reports. Questionnaires can be used to collect general data from respondents [21] or to validate measurements obtained by sensors [22]; and self-reports can be used to learn information regarding violations and accidents [23]. The primary motivator of some of the research of driver behavior analysis is that dangerous driving is a predictor of road accidents [24].

The advantages of using questionnaires are that they obtain personalized results and assign values to variables of interest. The variables’ values result from a series of responses, which together determine the value assigned to a variable. Thus, we do not ask users which value they would assign to a given variable; else, we derive the value of a variable from the answers to the questions that characterize the variable. The process of inferring the value of the variable from the answers to related questions avoids biases in the face of possible scenarios of social desirability. In behavioral sciences, studies related to traffic accidents found that the human behavior factor is the most critical variable in the process of driving a vehicle and its possible accident [8,25]. Researchers have developed self-applied tools (questionnaires) that aim to determine driving styles from the answers provided by drivers. Among the available questionnaires, some of the areas investigated are: classifying driving style [26], dangerous behavior [27], driving with a propensity to anger [28], driving with angry thoughts [29], and so on. However, the main shortcoming of these instruments in terms of reliability and accuracy is the social desirability of the respondents [30,31].

One way to provide reliability and formalism to drivers’ responses to questionnaires is through tools such as the Analytical Hierarchy Process (AHP), which is a support technique used in Multi-Criteria Decision Analysis (MCDA) [32]. The purpose of this technique is to support subjective evaluations, determining the relative importance between criteria by employing Saaty’s pairwise scale (see Table 1) that allows them to be ranked [33]. AHP employs pairwise comparison processes, hierarchical ranking, and calculating importance weights [34]. AHP is used to represent the decision by establishing hierarchies. However, designing hierarchies requires experience and knowledge of the situation to be solved, therefore it is suggested to have experts’ knowledge or subject information. In AHP, criteria priorities are obtained by comparing the importance pairwise concerning the goal [35,36,37]. Given the reliability of multicriteria tools in decision making, AHP is used in this research to formalize subjective information from perceptions. The AHP has proven its effectiveness in several areas [38], with vehicle driving safety being one example [39].

As for proposals that capture the environment surrounding the vehicle and the driver, in the approach presented by [40] ambient stereoscopic images are used to predict future driver maneuvers some time before they occur, given information about the driving context. Ref. [41] did a comparative study machine learning techniques for lane change detection, both works with the limitations inherent to physical coverage that can be had with video cameras.

Approaches to assess driving behavior based only on the driver, the vehicle or the environment are incomplete proposals, as [42] concluded that driver behavior should be modeled and evaluated in terms of different dimensions established within a driver–vehicle–environment system [43]. That is, in terms of the driver, their physiological, psychological and social profile (including gender, age, education and driving history) should be considered; in terms of the vehicle, the circumstances to be observed are those where the vehicle characteristics will somehow influence driving behavior; in terms of the environment, the factors to be considered are road geometry, road condition, road type, weather condition, light condition and traffic condition.

### 2.2. Fuzzy Systems

The evaluation of dangerous driving is performed in a scenario that involves vagueness and imprecision, which is why fuzzy systems are useful in addressing ambiguous conditions through fuzzy logic theory. The theory of fuzzy logic is inspired by the processes of human perception and cognition. This theory assigns to a variable instance a certain membership degree to a group from the set of fuzzy groups, representing the universe of discourse for that variable [44]. Fuzzy logic can deal with uncertain, imprecise, vague, partially true or unbounded information arising from perception and cognition.

Fuzzy logic provides an effective means for multi-criteria conflict resolution and a better evaluation of alternatives. Fuzzy logic-based systems can build intelligent systems for decision-making [45] that include vague human evaluations [46] and these systems have been employed to detect dangerous driving [47] and in general for automotive engineering applications [48]. The benefits of fuzzy models are well supported, for instance, in [49] the authors mention that fuzzy control is a nonlinear control technique that is relatively easy to understand and transparent with respect to other nonlinear techniques since it incorporates the knowledge and experience of the designer.

The approach presented in [50] is the closest to the one we propose. The authors used triaxial accelerometers’ data to feed a fuzzy system for pronation and supination assessment in Parkinson’s disease. Then, they contrasted the result of the system against experts’ opinions. Thus, the authors used the AHP to apply it to the experts’ assessments and compare the experts’ results against the results provided by the sensors. However, they did not use expert experience or patient opinions/feedback to feed the fuzzy system in the classification process.     

## 3. Reference Model

The reference model is a fuzzy system that allows combining objective and subjective input variables. Objective values are obtained from readings made with sensors of various types, e.g., motion sensors, temperature, turns, location, etc. On the other hand, subjective values are captured from the answers to questionnaires applied to users; the questionnaire or group of selected questionnaires will depend on the specific scenario and application. Perceptions, collected from users through questionnaires’ answers, are formalized using a method that facilitates the quantification and value (hierarchization/classification) given to the subjective data. Conventionally, fuzzy systems use quantitative input variables; however, there are scenarios that should be modeled by a system that additionally has qualitative input variables.

**Figure 1:**
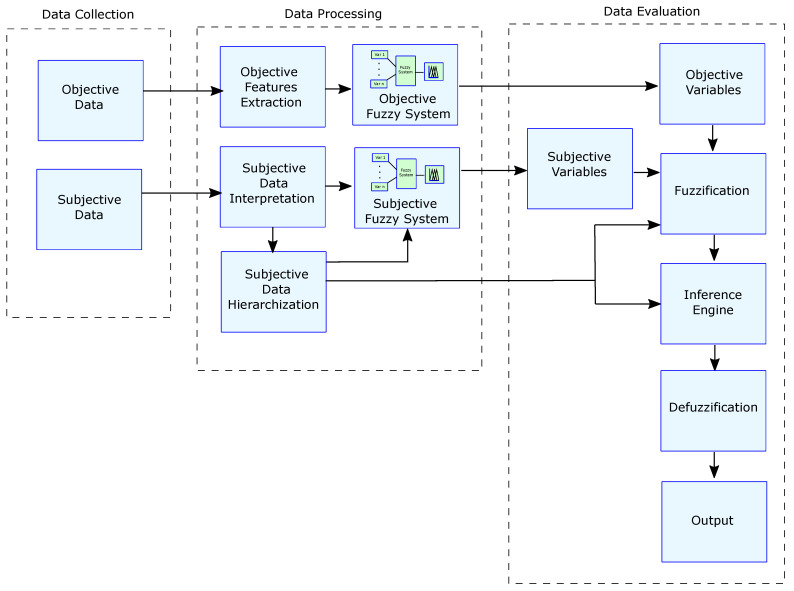
Reference model.

The proposed model offers a way to “convert”, by an appropriate method, the subjective values into objective ones so that they can be quantified and fed to the fuzzy system. The result is a fuzzy system model more in line with reality, where objective and subjective variables are involved. The purpose of the model is to address a classification process considering objective and subjective variables. The model comprises three phases: (i) data collection, (ii) data processing and (iii) data evaluation (see Figure 1).

### Case Study

In the present study, we decided to use the DDDI questionnaire because it is oriented to assess dangerous driving according to three dimensions: (i) Aggressive Driving (AD), (ii) Negative Cognitive/Emotional Driving (NCED), and (iii) Risky Driving (RD). These dimensions are relevant given the focus of the research, which aims to complement the data from the sensors considered (accelerometer, gyroscope, speedometer) with expert knowledge (obtained from the questionnaire). That is, the sensors considered can measure real physical actions (sharp turns, sudden braking, steering or lane deviations, racing) resulting from aggressive driving and speeding over bumps and potholes related to risky driving. Therefore, there is a correspondence between the data collected from the sensors and the specific dimensions evaluated by the DDDI questionnaire.

Considering the areas of technology and behavioral sciences briefly described in the previous paragraphs, the main objective of this paper is to develop a model for the evaluation of dangerous vehicle driving that uses, as a source of data, the measurements of vehicle motion sensors and the answers to questionnaires that rate the driver’s reactions to driving. The novelty of the method consists in the union of objective and subjective measurements.

## 4. Model Implementation in the Case Study

The implementation of the model requires a three-phase or stage process: (i) data collection, (ii) data processing, and (iii) data evaluation.

**Figure 2:**
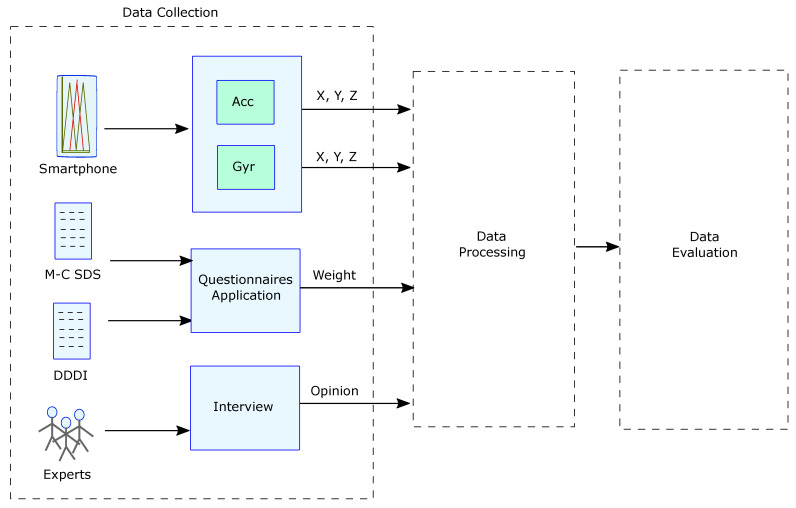
Data collection stage.

The first stage of the model contemplates obtaining objective data through sensors, which in other studies have proven to be effective with this type of measurements [51]. We perform several tasks within this first stage, ranging from selecting the route that meets the requirements to evaluate vehicular driving in a real environment, to collecting the data obtained in a natural environment (see Figure 2).

In this same stage, we also obtained subjective data from the results of the application of questionnaires to users. Questionnaires have proven to be effective in measuring people’s behavior in various domains [26,27,28,29]. In this same phase, we collected the opinions and experiences of mobility experts regarding the importance of each dimension of the DDDI questionnaire concerning the others. We used this expertise in phase two.

For the present investigation, the DDDI was used to assess dangerous driving behaviors, and the Marlowe–Crowne Social Desirability Scale (M-C SDS) was applied to know social desirability bias from drivers. As a result, we obtained qualitative answers of the driver’s driving behavior with the DDDI and the degree of reliability of the driver’s responses with the M-C SDS.

**Figure 3:**
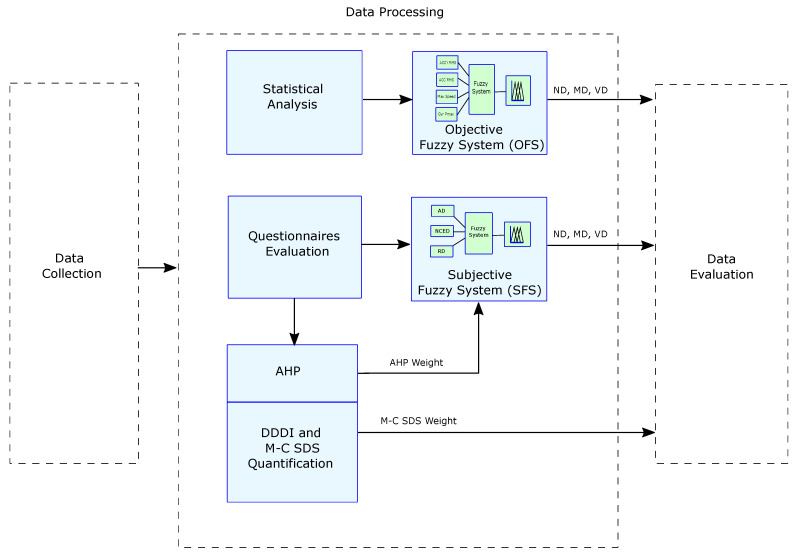
Data processing stage.

In the second stage of the study, we converted the objective values obtained by the sensors to statistical measurements similar to those considered in related studies [52,53,54]. Then, the most relevant features were selected to characterize the particular vehicular driving event. The chosen statistical values were input variables of an Objective Fuzzy System (OFS) that classified vehicular driving as: “Not dangerous”, “Moderately dangerous”, or “Very dangerous”, considering only objective data (see Figure 3).

We formalized the DDDI responses by quantification resulting in three weights corresponding to the aggressive, risky and negative cognitive/emotional driving in this same stage. The values of the dimensions represent the input variables of the Subjective Fuzzy System (SFS).

Then, according to the experts’ opinions given in the first phase, we ranked the subscales (aggressive, risky and negative cognitive/emotional driving) using AHP. In this case, we applied AHP to determine the importance of each of the DDDI dimensions. This is because although AHP is used to assign weights to criteria and select the best alternative, it can also be used only to assign weights to the dimensions of the questionnaire [33,55], as is done in our case. Moreover, we built the rules using the ranking obtained with AHP. The result of the SFS was the level of dangerous driving considering only subjective data.

The answers were interpreted and then ranked into low and high desirability weights with respect to the M-C SDS questionnaire. Finally, we used the results from the M-C SDS in the inference rules of the Combined Fuzzy System (CFS).

The third stage consisted of a fuzzy system that has the output of the OFS fed with sensor data as a first input variable. The second input variable is the result of the SFS.

**Figure 4:**
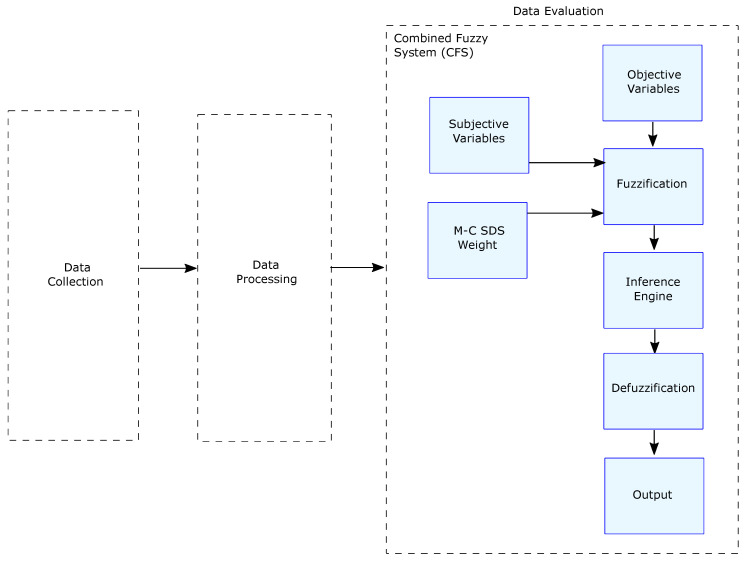
Data evaluation stage.

Once the values were fuzzified, we strengthened the inference engine with the desirability weights obtained from the M-C SDS questionnaire. The weights are part of the rules and are used to give credibility to the CFS input variables when they present discrepancies. The result of the CFS will be the final hazard level given to the driving (see Figure 4).

**Figure 5:**
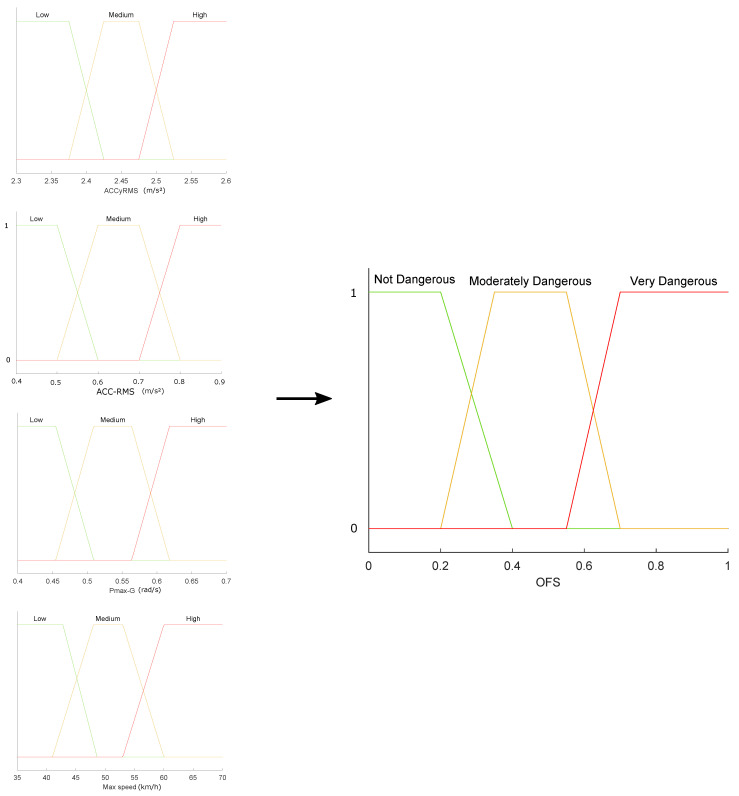
OFS Membership functions diagram.

**Figure 6:**
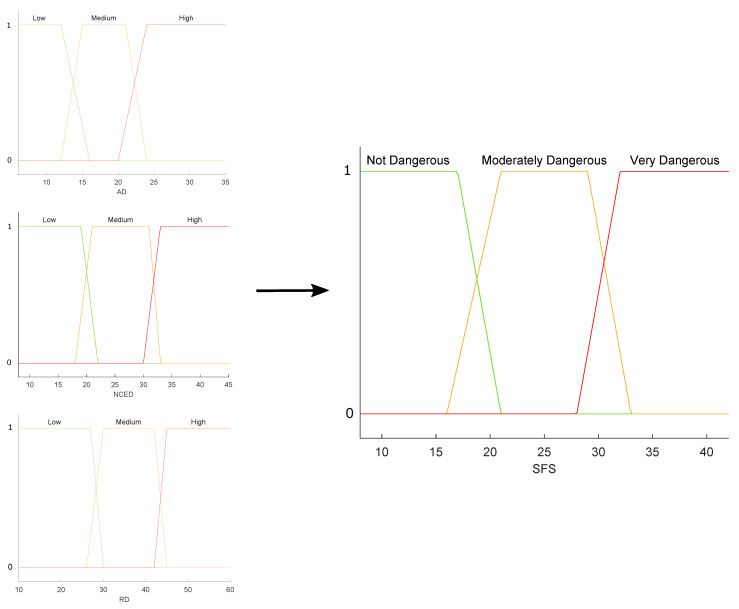
SFS Membership functions diagram.

**Figure 7:**
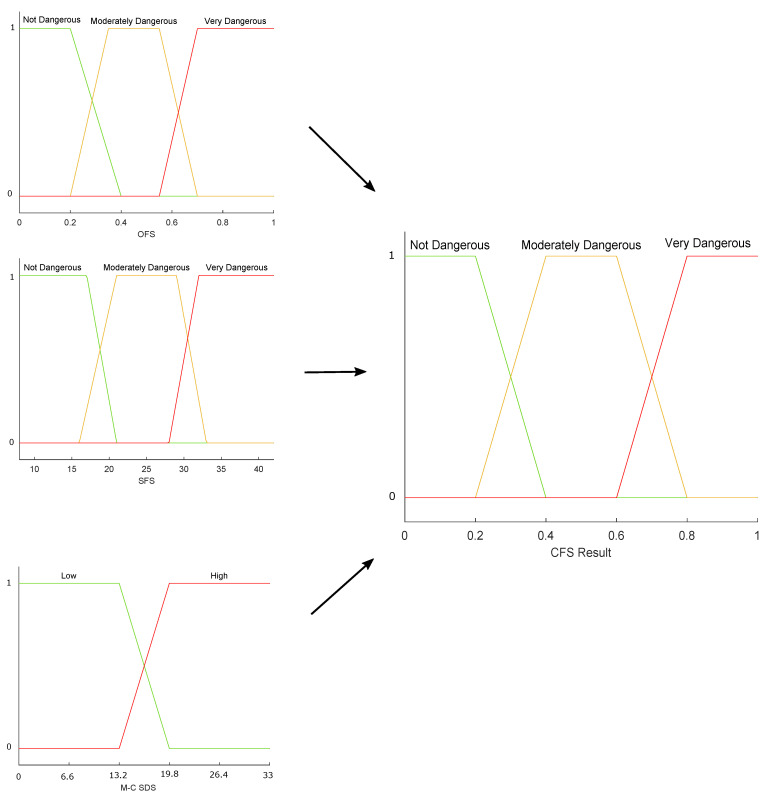
CFS Membership functions diagram.

A diagram of all the membership functions used in the fuzzy systems that integrate the proposal is shown in Figure 5, Figure 6 and Figure 7. From a set of characteristics obtained to feed the fuzzy systems (OFS and SFS), we analyzed and selected those characteristics with values that presented more variability among them, i.e., that there were significant differences between the values obtained for each characteristic. Then, according to the maximum and minimum values recorded for each variable, the universe of discourse (domain) was determined. It was decided that the shape of the membership functions should be trapezoidal because the maximum value of membership (one) applied to a set (range) of discrete values of the variables; therefore, trapezoidal functions are adequate [49] to represent the case study.

In a first approximation, the universe of discourse was symmetrically divided into three sets, since such a number of sets is usually common in fuzzy systems. The percentage of overlap between two neighboring sets was between 10% and 50%, ensuring that the sum of the overlap membership values was not greater than unity. These groupings made it possible to associate each input linguistic value with its respective fuzzy set. The ranks and overlaps of the sets were optimized based on expert opinion.

The Algorithm 1 contains the steps to build the whole OFS subsystem. The membership functions for the OFS subsystem are shown in Figure 5.

The Algorithm 2 contains the steps to build the whole SFS subsystem. The membership functions for the SFS subsystem are shown in Figure 6.
**Algorithm 1** Driver classification (OFS).**Input:** Raw data **Output:** Objective driving behavior classification  1:Load raw data from sensors (accelerometer, gyroscope, and speedometer)  2:Compute features
(1)$$RMS=\sqrt{\frac{1}{n}\sum_{i=1}^{n}x_{i}^{2}}$$
(2)$Max\;peak\;(x_{i})$  3:Select relevant features  4:Define rules if RMS(x) is (Low/Medium/High) and/or AccY-RMS is (Low/Medium/ High) and/or GyrPmax is (Low/Medium/High) and/or Max speed (Low/Medium/ High) then OFS is (Not Dangerous/Moderately Dangerous/Very Dangerous)  5:Fuzzify (Design the membership groups). Trapezoidal function define a,b,c,d
(3)$$f\left(x,a,b,c,d\right)=\{\begin{array}{c} 0,x\leq a\;\\ (x-a)/(b-a),a\leq x\leq b\;\\ 1,b\leq x\leq c\;\\ (d-x)/(d-c),c\leq x\leq d\;\\ 0,x\geq d \end{array}$$  6:Evaluate from rules  7:Defuzzify
(4)$xcentroid=\frac{\sum_{i}\mu \left(x_{i}\right)x_{i}}{\sum_{i}\mu (x_{i})}$  8:If the classification accuracy > 70%, go to step 10  9:Define membership group adequacies and go to step 510: End of OFS classification


**Algorithm 2** Driver classification (SFS).**Input:** data from answers to questionnaires **Output:** Subjective driving behavior classification
  1:Load data  2:Compute subjetive features from-   Hierarchize the DDDI dimensions (AHP)-   Hierarchize social desirability (M-C SDS)  3:Define rules if AD is (Low/Medium/High) and/or NCED is (Low/Medium/High) and/or RD is (Low/Medium/High) then SFS is (Not Dangerous/Moderately Dangerous/Very Dangerous)  4:Fuzzify (Design the membership groups). Define a,b,c,d from Equation (3) for trapezoidal function.  5:Evaluate from rules  6:Defuzzify applying Equation (4)  7:End of SFS classification


The Algorithm 3 contains the steps to build the whole CFS subsystem. The membership functions for the CFS subsystem are shown in Figure 7.
**Algorithm 3** Driver classification (CFS).**Input:** output data from OFS, SFS and M-C SDS **Output:** Driving behavior classification  1:Load data  2:Define rules if OFS is (Not Dangerous/Moderately Dangerous/Very Dangerous) and/or SFS is (Not Dangerous/Moderately Dangerous/Very Dangerous) and/or M-C SDS is (Low/High) then CFS is (Not Dangerous/Moderately Dangerous/Very Dangerous)  3:Fuzzify (Design the membership groups). Define a,b,c,d from Equation (3) for trapezoidal function.  4:Evaluate from rules (weighting M-C SDS)  5:Defuzzify applying Equation (4)  6:If the classification accuracy > 70%, go to step 8  7:Define membership group adequacies and go to step 3  8:End of CFS classification


The construction of the rules was based on the experts’ opinion. According to the experts, all possible combinations for the variables involved in the fuzzy models were considered, that is, the multiplicative rule was applied. A total of 81 rules (3x3x3x3) were constructed for the OFS model, 27 rules (3x3x3) for the SFS model and 18 rules (3x3x2) for the CFS model. The output value for each combination was determined by the experts. All the rules constructed are necessary as they are required to cover all the driving scenarios studied, according to expert opinion. The defuzzification method used was the centroid method.

### 4.1. Empirical Evaluation of the Solution

The experimental design contemplated aspects such as invitation of potential participants, selection of volunteers, choice of geographic location for the tests, placement of the devices inside the vehicle, selection of sensors, applications used, evaluation of the data, among others.

### 4.2. Participants

Participants consisted of a heterogeneous group of 19 drivers (5 women and 14 men). The age range was between 17 and 67 years (mean = 43.42). The driving experience was in the range of 1 to 20 years. Thus, the sample size used in the present investigation proved to be adequate for this type of study [56]. We provided participants information about the research, including a brief explanation of its purpose. We assured them their information would be anonymous, and confidential. All subjects provided written informed consent prior to participating in the study.

### 4.3. Chosen Route

We chose a 3.6 km long circuit located on an avenue in the city of Villahermosa, Tabasco, Mexico. The circuit has bumps, potholes, traffic lights and maximum speed indicators; the road has the right conditions to observe possible dangerous behaviors during vehicular driving. During the experiments, the selected route was traveled twice by each driver. This was for each driver to become accustomed to the vehicle and to avoid biases in the measurements. The measurements collected from the second time the route was completed were considered for data analysis.

### 4.4. Vehicle and Smartphones

The vehicle used was a 2005 model common-use vehicle. We made tests to select the best position for the sensors considering the center of mass of the vehicle in order to obtain more reliable readings [57]. Inside the vehicle, we made the necessary preparations to install three smartphones very well fastened. The first phone was placed close to the vehicle’s center of mass and gathered readings from the acceleration, gyroscope and GPS sensors. The second phone was placed between the steering wheel and the vehicle’s speedometer and recorded video of speedometer variations during driving. Finally, the third phone was attached to the pickup’s front rearview mirror and collected: video of the street the driver was driving on, acceleration patterns when starting gears and brake application patterns when stopping the vehicle.

The technical specifications of two of the cell phones used are: Huawei Y6, model MRD-LX3, Android 9 version, RAM 2.0 GB, with a total storage capacity of 32 GB (internal storage). The third phone was Huawei Y9s, model STK-LX3, Android 10 version, RAM 6.0 GB, and a total storage capacity of 128 GB.

The X, Y and Z-axis readings were taken at a sampling rate of 100 Hz for accuracy, although previous studies have shown that 50 Hz is sufficient [58,59].

### 4.5. Route Video

**Figure 8:**
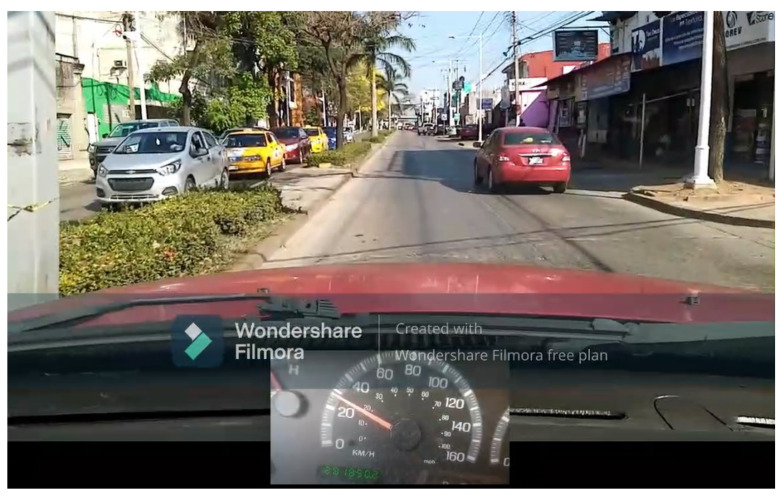
Road way and speed video.

We recorded the entire route on video with a camera aiming at the front of the vehicle and the car’s speedometer so it could be observed, for example, if the driver braked abruptly or accelerated too much when starting the car. The purpose of recording the route was so that experts could evaluate the driving by observation as a means of validation (see Figure 8).

### 4.6. Questionnaires

We applied two questionnaires to each driver; the first was the DDDI, and the second was the Marlowe–Crowne Social Desirability Scale (M-C SDS). Participants answered both questionnaires only once before or after the driving test.

The DDDI questionnaire is an instrument which assesses dangerous driving and consists, in its original version, of 28 items grouped into three subscales, which measure negative cognitive/emotional, aggressive and risky driving (examples: I drive when I am angry or upset; I will weave in and out of slower traffic; I make rude gestures). Each item is in the form of a statement which is answered with a Likert-type scale, ranging from (0) never, (1) almost never, (2) sometimes, (3) almost always and (4) always.

The M-C SDS is a self-report questionnaire that assesses whether respondents are concerned about social approval. The questionnaire measures the social desirability bias that may be contained in survey responses and represents a common bias affecting research [60].

The MC-SDS consists of 33 items, 18 of which (direct items) reflect socially desirable but infrequent behaviors and traits (e.g., I never hesitate to go out of my way to help someone in trouble), while the remaining 15 items (inverse items) reflect undesirable but widespread behaviors and traits (e.g., I like to gossip at times).

## 5. Results

This section presents the results for the three phases of the model: the data collection stage, data processing stage, and data evaluation stage.

### 5.1. Data Collection Stage

#### 5.1.1. Sensors’ Readings

In the tests conducted during the route, we gathered data from the accelerometer and gyroscope sensors. The number of records ranged between 53,000 and 111,000 for each user. The records variation was due to the time spent for the ride depending on traffic and driving style. The selected features obtained with the accelerometer were RMS of the Y-axis (AccY-RMS) and RMS of the vector sum of the axes (Acc-RMS); from the gyroscope, we chose the maximum peak of the vector sum of the axes (Gyr-Pmax), and finally, the maximum velocity (Max speed) was considered (see Table 2).

#### 5.1.2. Questionnaires Application

In this same stage, we collected the subjective data using questionnaires which were applied to the drivers before or after performing the vehicle driving test. The responses varied according to the Likert scale for the DDDI questionnaire, with values ranging from 1 (“Never”) to 5 (“Always”). DDDI-based classification was considered ND for scores between 28–56, MD for scores between 57–84 and VD for scores between 85–140 (see Table 3).

In the M-C SDS questionnaire, the user can answer the item with true or false and obtain a score. The sum of the item scores results in a total score between 0 and 33. A higher score indicates greater social desirability, which is understood as response bias or personality trait (defensiveness). For scores between 0–16, social desirability was considered Low, and between 17–33 as High (see Table 4).

#### 5.1.3. Expert’s Interview

We conducted a meeting with three experts in mobility to collect opinions on the importance of each dimension from DDDI. Then, we averaged the views and used them in the data processing stage.

### 5.2. Data Processing Stage

#### 5.2.1. Results Obtained with Sensors

**Figure 9:**
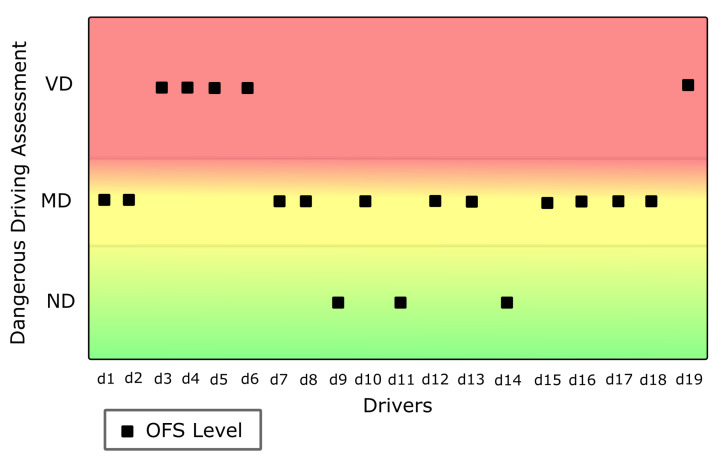
Objective Fuzzy System (OFS) results.

The variables AccY-RMS, Acc-RMS, Gyr-Pmax, and Max-speed fed the OFS subsystem. From 81 applied rules, sensor data-based categorization of dangerous vehicular driving was obtained. The results are as shown in Figure 9.

#### 5.2.2. AHP

**Figure 10:**
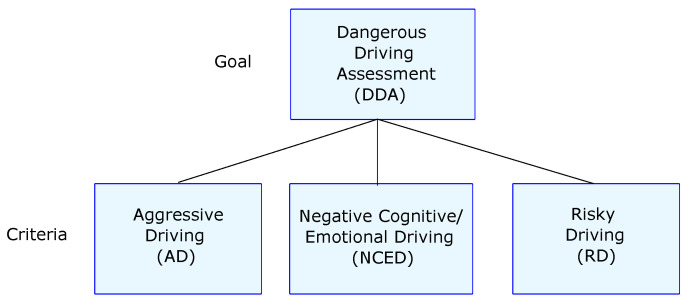
Two hierarchy levels.

In AHP, we set the evaluation of dangerous driving as the objective, and we considered the DDDI dimensions as criteria to generate the pairwise matrix (see Figure 10). Then, the mobility experts assigned the level of importance of each dimension concerning the others, using Saaty’s comparison scale [35]. The generated matrix can be seen in Table 5. Finally, we calculated the overall priorities or weights of the criteria (see Table 6) using the approximate method due to its simplicity [61].

With the priorities, we calculated the weights for each driver’s test (see Table 7).

To avoid incosistencies we checked the consistency ratio defined as *CR*, which is shown in Equation (5).

(5) $$CR=\frac{CI}{RI}$$

where *CI* is consistency index and *RI* is the consistency index of a random-like matrix.

*CI* is calculated as shown in Equation (6).

(6) $$CI=\frac{\lambda_{max}-n}{n-1}$$

where *n* is the number of compared elements.

($\lambda_{max}$) is calculated as shown in Equation (7).

(7) $$\lambda_{max}=\frac{\sum_{\left(WeightedSum)(Priority\right)}}{n}$$

Considering the values *n* = 3 (dimensions), $\lambda_{max}$ = 3.008 and *RI* = 0.58 (according to Table 8, for *n* = 3, *RI* = 0.58), we obtained the value of the Consistency Ratio [39,61].

The Consistency Ratio value was 0.007 (0.004/0.58), and since it is less than 0.10, the pairwise matrix is reasonably consistent [61].

#### 5.2.3. Subjective Fuzzy System results

**Figure 11:**
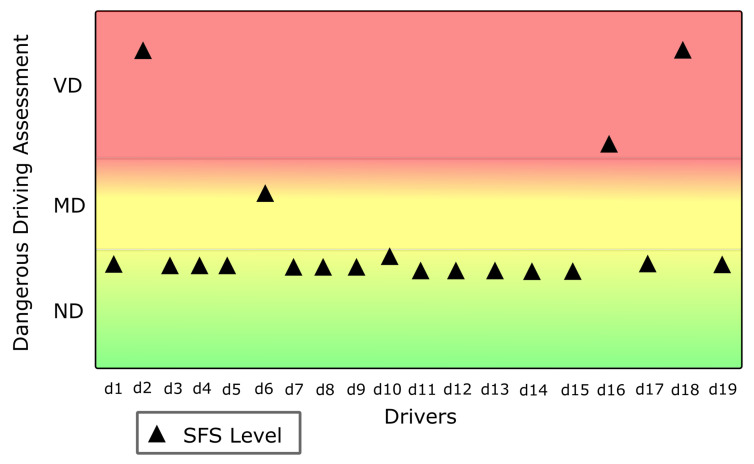
Subjective Fuzzy System (SFS) results.

The results obtained from evaluating the questionnaire with the Subjective Fuzzy System (SFS) are presented in Figure 11.

### 5.3. Data Evaluation Stage

**Figure 12:**
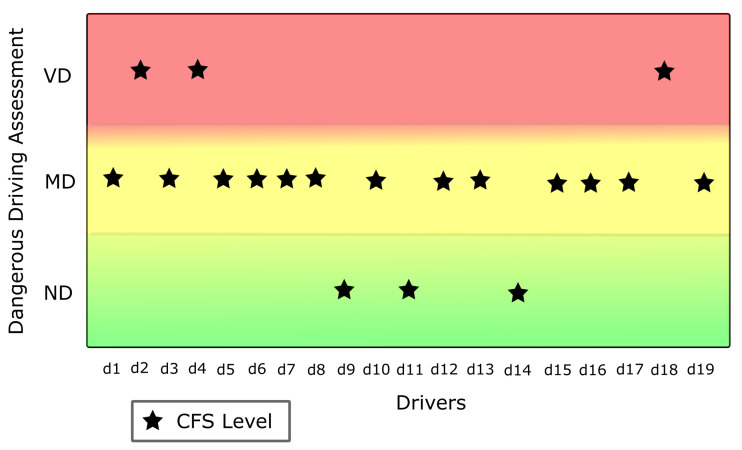
Classes from Combined Fuzzy System (CFS).

In this stage, we implemented a Combined Fuzzy System (CFS), taking the results of the OFS and SFS systems as input variables. We used the result of the M-C SDS questionnaire in the inference engine to determine the weights of the objective variable, derived from OFS, and of the subjective variable from SFS. We combined both variable types in this CFS, and the driver’s social desirability was the third crucial variable for final categorization. The numerical results are shown in Table 9 and the derived classes are illustrated graphically in Figure 12.

### 5.4. Validation

**Figure 13:**
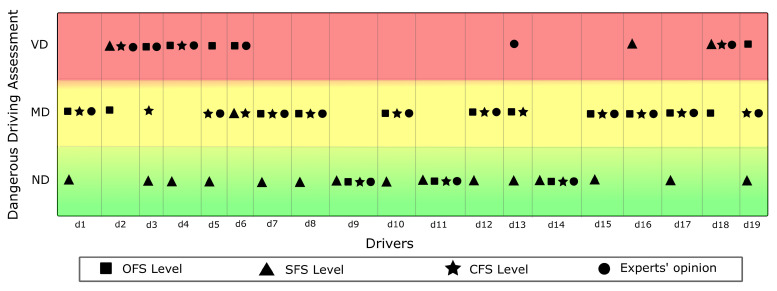
Comparisons against Expert opinion.

Three mobility experts validated the efficiency of our proposal, the Combined Fuzzy System (CFS). For this purpose, the experts viewed and analyzed the video of the test drive for each driver, and we provided them with the results of the DDDI and M-C SDS questionnaires answered by the driver. Based on the above, the experts consensually classified each driver’s level of danger (ND, MD, and VD). Finally, we present the result of the validation in Figure 13.

From the results obtained and further calculations, we have a 73.68% of coincidence in classification, respect to experts opinion, when only considering the use of sensor data to feed the fuzzy system (OFS). On the other hand, if the fuzzy system is fed only with the answers from the questionnaires (SFS), the coincidence achieved is only 26.31%, which we attribute to the high social desirability of most drivers evaluated and which is in line with the results shown in Table 4. However, an agreement of 84.21% was achieved from the combined fuzzy system (CFS), concerning the experts’ evaluation. This demonstrates the benefits of the multidisciplinary approach to achieve a classification more in line with the experts’ evaluation.

**Figure 14:**
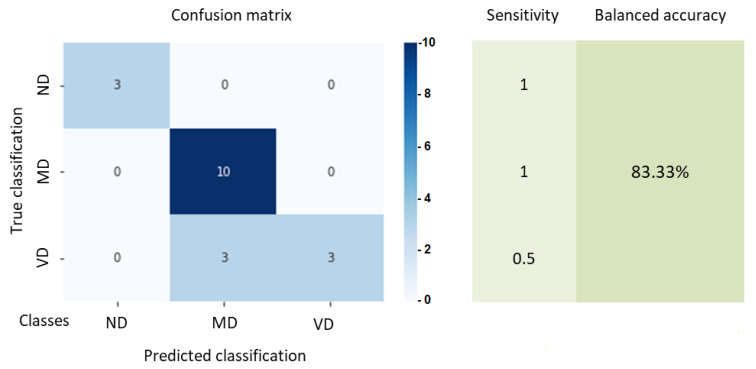
Confusion matrix, sensitivity and balanced accuracy.

Figure 14 shows the confusion matrix between the true classification from expert opinion and the classification predicted by our proposed approach. Statistical metrics (sensitivity, balanced accuracy) that are appropriate for unbalanced multiclass classifications are also shown. The latter also confirms the efficiency of the presented proposal. Although the sensitivity value obtained to correctly classify VD driving represents a major drawback.

The results obtained reflect the knowledge contained in the theory. That is, the sensors used (accelerometer and gyroscope) were able to measure vehicle movements, although in some cases the values obtained were erroneous. Regarding the questionnaires used to obtain subjective information from the drivers, although the drivers freely expressed their opinions, the results were biased by the social desirability present in several cases. The fuzzy systems, on the other hand, modeled the ambiguities of the variables involved and performed a mostly accurate classification, after defuzzification.

## 6. Discussion

The most significant achievement of our proposed approach is to have demonstrated that a multidisciplinary-based fuzzy system can evaluate dangerous driving, using the strengths of each discipline and compensating the limitations of the disciplines involved. The proposed three-phase fuzzy system includes input variables and inference rules, objective values from electronic engineering (sensors), and subjective values from behavioral science (answers to questionnaires). Both types of values were processed computationally to generate categorization values with an efficiency higher than 84%. This efficiency is outstanding as it is an experiment conducted in a real scenario with no control over traffic. Additionally, the drivers who performed the driving sessions had complete freedom of style without predefined events. To our knowledge, this is the first approach that combines the selected disciplines to evaluate dangerous driving and allows an evaluation from a holistic approach.

The efficiency of the CFS was validated with expert opinions from observing videos of experiments, and from the results of questionnaires. Expert opinions have been used to validate experiments in real scenarios [62].

Our findings are well substantiated by similar studies divided into two major groups. The first group of studies employed artificial intelligence and electronic devices with embedded sensors, having the disadvantages of failures and errors in readings [63], predefined and controlled events, and simulated scenarios, where sensors were used. In the second group are the technological studies (sensors and fuzzy systems), which are supported by self-reports, although the latter are not combined within the fuzzy system, they are only used to categorize groups or to validate the results.

An example of the first group of studies is an investigation in which an application based on smartphone sensors and supported by a fuzzy system was used to classify aggressive driving in five groups. To validate their results, users were asked to evaluate themselves by answering a question, where they had to indicate how they considered their aggressive driving had been, on a scale of one to five. Only in 60% of the cases there was a coincidence in the users’ self-assessment and fuzzy system classification [10].

There was another study that used supervised machine learning and fuzzy logic to detect aggressive events such as aggressive braking, aggressive acceleration, aggressive turning, aggressive lane changes and non-aggressive events. In this work, the events were controlled, since they were already defined, and an efficiency between 98 and 99% was achieved. However, the drivers were not free to drive as they would have in a real environment [59].

Another work obtained a lower efficiency than our proposed approach, using a smartphone and neural networks, achieving 77% efficiency in the classification of driving styles such as aggressive, normal and quiet [19]. Unlike our approach, this one used an OBD-II device for data capture, which substantially increased the equipment costs because it was an additional piece of equipment to the sensors embedded in the smartphone (GPS and accelerometer in this case).

In the second group of studies supported by self-reports, there is an approach [64] that proposes a neuro-fuzzy system to classify driving behaviors taking into account similarities with fuzzy patterns of driving maneuvers. They recognized driving maneuvers including lane changes, left or right turns and U-turns. The validation of that approach was performed with the Driver’s Angry Score (DAS) questionnaire, obtaining an efficiency of 87% [64].

Another study, which also employed self-reports, analyzed driver behavior using a hybrid of Discrete Wavelet Transformation (DWT), Adaptive Neuro Fuzzy Inference System (ANFIS) and smartphone sensors (accelerometer, gyroscope and magnetometer). The categorization included the following behaviors: safe, semi-aggressive, and aggressive. These three classes of behaviors have been extracted from the Driver Anger Scale (DAS) self-reported questionnaire. The DWT was used to assess features, validating the results with the DAS questionnaire and obtaining an efficiency of 84.2% on average [65]. In this case, their efficiency results are similar to the ones we obtained.

Our approach is in line with other studies where it is established that hybrid solutions that combine data sources produce more reliable and accurate results, particularly in assessments related to driving performance [62,66].

In the specific case of the present research, the fact that the tests were personalized allowed us to have adequate results regardless of the number of users. Personalized results were sought, as is the case with the questionnaire, where personality is reflected. No attempt was made to recognize a generalized pattern of a broad population.

There were some limitations during the driving tests; for example, because the experiment was in a real scenario, it was complex to ensure that traffic conditions were very similar for each test, despite the fact that the same day and time was established. Similarly, we could not capture some situations with the implemented sensor design, i.e., additional devices (weather sensors, proximity sensors, and cameras for a 360-degree approach) would be needed to capture the entire driving environment.

In addition, a limitation of questionnaires is the known social desirability that may arise when drivers provide answers according to current societal norms and values [67]. As stated in [68], self-report measurements of driving behavior, the same as all self-report measurements, are subject to respondent recall error as well as information validity (including social desirability bias). Additionally, employing questionnaires where responses are based on past behavior may be obsolete, as certain personal conditions of the driver may change from day to day [69]. Nevertheless, another situation that could have arisen during the driving tests, which is inherent to human beings, is the drivers’ attitude change when they know they are being evaluated [70].

During the development of the research we encountered some difficulties. For example, properly holding the smartphones to capture the movements, i.e., that the captured movements were not the result of improper holding. Additionally, during sensor measurements, in at least two cases, we had erroneous readings.

The proposed approach is innovative because it addresses the need to evaluate dangerous driving from a multidisciplinary perspective. Multidisciplinarity is relevant for solving complex social problems [71,72]. The fuzzy system presented is an efficient alternative for decision-making that can help to increase safety in intelligent transportation systems.

## 7. Conclusions

The proposed system represents an advancement in the current state of knowledge since it addresses the need to evaluate vehicular driving from a multidisciplinary approach. The materials and methods used are of proven reliability and efficiency in each discipline involved. Furthermore, we combine the methods within a fuzzy system that allows handling the inherent vagueness when the criteria’ values are not precise. Additionally, subjective variables contribute to evaluating drivers in a personalized way.

The system’s categorization is the result of considering both objective (sensors) and subjective (questionnaires) aspects of driving behavior, thus considering the diversity of factors that intervene during the driving process. Therefore, we fulfill the objective of having a system that combines objective and subjective variables, and we address the absence of a combined system for the evaluation of dangerous driving.

Our model aims to take advantage of the benefits of each tool, compensating the disadvantages and minimizing biases, in order to increase efficiency and reliability when assessing dangerous driving. We found that the combination of disciplines to assess driving behavior makes the accuracy of the classification closer to the assessment made by experts than if the behavior were assessed only objectively or subjectively. Some possible uses of the proposed approach are: as a tool for driving personnel selection, driver assistant and monitoring of driving styles and behaviors.

Future work on this approach may include increasing the variety of sensors to capture driving conditions, such as more video cameras, weather sensors, proximity sensors, as well as physiological information (electrocardiogram, electro dermal activity and respiration), and facial recognition sensors (for fatigue and distraction detection). The inclusion of more users and experiments to strengthen the validity of the proposal is convenient. Related to fuzzy logic theory and with the purpose of improving the proposal, we can consider the use of type-2 fuzzy systems for uncertainty management, as well as type-3 fuzzy systems [73]. Another improvement opportunity for this approach is automatic optimal tuning using metaheuristic optimization algorithms [49].

## Figures and Tables

**Table 1:** Saaty’s pairwaise scale.

Verbal Judgment	Numeric Value
Extremely important	9
	8
Very strongly more important	7
	6
Strongly more important	5
	4
Moderately more important	3
	2
Equally important	1

**Table 2:** Selected features from sensors.

Driver	AccY-RMS	Acc-RMS	Max Speed	Gyr-Pmax	Output
d1	2.4756	0.6586	52	0.5379	0.4500
d2	2.4155	0.6636	50	0.6011	0.6311
d3	2.4252	0.6248	60	0.5706	0.8084
d4	2.5366	0.8762	60	0.5497	0.8125
d5	2.3200	0.6100	69	0.6800	0.8130
d6	2.4534	0.6802	63	0.6397	0.8125
d7	2.4490	0.6810	47	0.5806	0.5564
d8	2.4405	0.6162	46	0.5056	0.4340
d9	2.4369	0.6403	42	0.4529	0.2008
d10	2.4449	0.6558	52	0.4573	0.4500
d11	2.3472	0.6867	40	0.4710	0.1650
d12	2.3984	0.6639	40	0.4781	0.3140
d13	2.5007	0.6666	51	0.5176	0.4500
d14	2.3398	0.5848	40	0.5119	0.1588
d15	2.4224	0.7091	47	0.4739	0.3838
d16	2.5802	0.4413	48	0.5794	0.4242
d17	2.4036	0.7017	46	0.5110	0.3492
d18	2.4039	0.7397	58	0.5712	0.5708
d19	2.4890	0.5850	46	0.7970	0.6540

**Table 3:** DDDI responses.

Driver	AD	NCED	RD	Score	DDDI-Based Classification
d1	10	23	19	52	ND
d2	27	36	47	110	VD
d3	9	18	17	44	ND
d4	9	13	12	34	ND
d5	9	14	14	37	ND
d6	14	30	25	69	MD
d7	7	16	14	37	ND
d8	7	11	12	30	ND
d9	7	18	15	40	ND
d10	7	20	18	45	ND
d11	8	17	15	40	ND
d12	9	16	14	39	ND
d13	7	23	17	47	ND
d14	8	16	16	40	ND
d15	11	15	14	40	ND
d16	19	28	19	66	MD
d17	12	11	15	38	ND
d18	26	37	45	108	VD
d19	7	11	12	30	ND

**Table 4:** M-C SDS responses.

Driver	Score	Social Desirability Level
d1	17	High
d2	17	High
d3	11	Low
d4	22	High
d5	16	Low
d6	16	Low
d7	31	High
d8	16	Low
d9	25	High
d10	27	High
d11	16	Low
d12	19	High
d13	22	High
d14	29	High
d15	23	High
d16	23	High
d17	17	High
d18	4	Low
d19	29	High

**Table 5:** DDDI pairwise matrix.

DDA	AD	NCED	RD
AD	1	7	3
NCED	0.143	1	0.333
RD	0.333	3	1

**Table 6:** Subscales’ priorities.

DDA	AD	NCED	RD	Priorities
AD	0.678	0.636	0.692	0.669
NCED	0.097	0.091	0.077	0.088
RD	0.226	0.273	0.231	0.243

**Table 7:** DDDI responses weighted.

Driver	Weighted
d1	13.331
d2	32.652
d3	11.736
d4	10.081
d5	10.655
d6	18.081
d7	9.493
d8	8.567
d9	9.912
d10	10.817
d11	10.493
d12	10.831
d13	10.838
d14	10.648
d15	12.081
d16	19.792
d17	12.641
d18	31.585
d19	8.567

**Table 8:** Consistency indices for random matrices.

*n*	1	2	3	4	5	6	7	8
RI	0.00	0.00	0.58	0.9	1.12	1.24	1.32	1.41

**Table 9:** Numerical results for Combined Fuzzy System (CFS).

Driver	M-C SDS	CFS Result
d1	0.5151	0.3960
d2	0.5151	0.8282
d3	0.3333	0.5000
d4	0.6666	0.8470
d5	0.4848	0.3479
d6	0.4848	0.6038
d7	0.9393	0.5199
d8	0.4848	0.3479
d9	0.7575	0.0153
d10	0.8181	0.5000
d11	0.4848	0.1702
d12	0.5757	0.4094
d13	0.6666	0.5000
d14	0.8787	0.1530
d15	0.6969	0.4835
d16	0.6969	0.5000
d17	0.5151	0.3960
d18	0.1212	0.8417
d19	0.8787	0.5000

## Data Availability

Not applicable.
